# Characterization of the metabolic shift between oxidative and fermentative growth in *Saccharomyces cerevisiae *by comparative ^13^C flux analysis

**DOI:** 10.1186/1475-2859-4-30

**Published:** 2005-11-03

**Authors:** Oliver Frick, Christoph Wittmann

**Affiliations:** 1 Biochemical Engineering Institute, Saarland University, POB 151150, 66123 Saarbrücken, Germany

## Abstract

**Background:**

One of the most fascinating properties of the biotechnologically important organism *Saccharomyces cerevisiae *is its ability to perform simultaneous respiration and fermentation at high growth rate even under fully aerobic conditions. In the present work, this Crabtree effect called phenomenon was investigated in detail by comparative ^13^C metabolic flux analysis of *S. cerevisiae *growing under purely oxidative, respiro-fermentative and predominantly fermentative conditions.

**Results:**

The metabolic shift from oxidative to fermentative growth was accompanied by complex changes of carbon flux throughout the whole central metabolism. This involved a flux redirection from the pentose phosphate pathway (PPP) towards glycolysis, an increased flux through pyruvate carboxylase, the fermentative pathways and malic enzyme, a flux decrease through the TCA cycle, and a partial relocation of alanine biosynthesis from the mitochondrion to the cytosol. *S. cerevisiae *exhibited a by-pass of pyruvate dehydrogenase in all physiological regimes. During oxidative growth this by-pass was mainly provided via pyruvate decarboxylase, acetaldehyde dehydrogenase, acetyl-CoA synthase and transport of acetyl-CoA into the mitochondrion. During fermentative growth this route, however, was saturated due to limited enzyme capacity. Under these conditions the cells exhibited high carbon flux through a chain of reactions involving pyruvate carboxylase, the oxaloacetate transporter and malic enzyme. During purely oxidative growth the PPP alone was sufficient to completely supply NADPH for anabolism. During fermentation, it provided only 60 % of the required NADPH.

**Conclusion:**

We conclude that, in order to overcome the limited capacity of pyruvate dehydrogenase, *S. cerevisiae *possesses different metabolic by-passes to channel carbon into the mitochondrion. This involves the conversion of cytosolic pyruvate either into acetyl CoA or oxaloacetate followed by intercompartmental transport of these metabolites. During oxidative growth mainly the NAD specific isoforms of acetaldehyde dehydrogenase and isocitrate dehydrogenase catalyze the corresponding reactions in *S. cerevisiae*, whereas NADPH supply under fermentative conditions involves significant contribution of sources other than the PPP such as e. g. NADPH specific acetaldehyde dehydrogenase or isocitrate dehydrogenase.

## Background

The budding yeast *S. cerevisiae *is an important biotechnological organism *e.g. *for production of ethanol [1], recombinant proteins [2], antibiotics [3] or biomass [4]. Additionally it is relevant as a model system to study metabolism due to its high homology with other eukaryotic organisms [5,6]. This explains the high interest in understanding metabolic function and regulation in this organism. *S. cerevisiae *is able to perform simultaneous respiration and fermentation at high growth rates even under fully aerobic conditions [7,8]. This Crabtree effect called phenomenon is visualized in chemostat culture of *S. cerevisiae *by a shift from biomass formation towards fermentative products at increased dilution rates [8]. Also other yeast species show this behavior [9]. Despite many studies in the past [10-12], a clear and univocal explanation of this phenomenon has not yet been provided [13,14]. The Crabtree effect has important consequences for industrial processes aiming at the production of yeast biomass, where the formation of fermentative by-products is undesired [15,16]. A powerful approach to investigate metabolic systems is ^13^C metabolic flux analysis which allows the quantification of *in vivo *activity of pathways and reactions [17-19]. In recent studies, which took the spatial arrangement of metabolic reactions in the compartments cytosol and mitochondrion into account, metabolic flux analysis was successfully applied to *S. cerevisiae *and related yeast strains [20-27]. In the present work we investigated the metabolic shift from oxidative to fermentative growth in *S. cerevisiae *by quantifying metabolic fluxes through the underlying reactions in the central metabolism at these different physiological states. For this purpose chemostat cultures with [1-^13^C] glucose as substrate were grown at different growth rates under aerobic glucose-limited conditions to metabolic and isotopic steady-state. The use of a continuous culture hereby allowed the precise adjustment of the physiological state of the cells, *i.e. *the relative activity of the fermentative pathways.

## Results

### Growth and product formation of *S. cerevisiae*

In order to identify the exact experimental conditions required to establish the physiological states of interest, chemostat cultures were carried out at different dilution rates between 0.10 h^-1 ^and 0.45 h^-1 ^(Figure 2). *S. cerevisiae *exhibited the typical growth behavior for this organism in continuous culture, characterized by a transition from purely oxidative metabolism at low growth rate to fermentative metabolism at high growth rate. At a growth rate below μ = 0.20 h^-1 ^the metabolism of *S. cerevisiae *was purely oxidative, indicated by the absence of fermentation products and a high biomass yield. At higher growth rates the Crabtree effect was induced, which is shown by enhanced formation of ethanol and acetate. Hereby, the relative contribution of fermentation to the overall metabolic activity increased with increasing growth rate. At μ = 0.30 h^-1 ^about 25 % of the utilized glucose was directed towards fermentation, indicating a still mainly respirative metabolism. Towards higher dilution rates the fraction of glucose channeled into fermentative pathways increased up to a value of more than 50 % at of μ = 0.40 h^-1^.

### Tracer experiments with [1-^13^C] glucose as sole carbon source

Selected chemostat cultivations with [1-^13^C] glucose as substrate were carried out for the elucidation of metabolic fluxes. Hereby, three distinct dilution rates, representing purely-oxidative (0.15 h^-1^), mixed respiro-fermentative (0.30 h^-1^), and mainly fermentative metabolism (0.40 h^-1^) were selected for the flux studies. The resulting ethanol yield under these conditions was 0.0, 0.5, and 1.1 mol ethanol (mol glucose)^-1^, respectively. The stoichiometric data obtained from the ^13^C cultures agreed well with the corresponding values from the initially performed cultivations (Table 3). The major by-products were ethanol, acetate and glycerol. In all cases the carbon balance was almost closed underlining the high consistency of the data. Labeling patterns of amino acids from cell protein, analyzed after 5 residence times, remained constant over 4 samples taken in 1 h intervals from the culture, i. e. the relative error for the mass isotopomer fractions was typically below 1 %. This indicated that the cells were in isotopic steady-state. To quantify metabolic fluxes a ^13^C flux model was applied that involves metabolite and isotopomer balancing. The used model fully considers specific features of the underlying metabolism, *e. g. *bidirectional reactions, label scrambling in symmetric molecules, ^13^C incorporation from CO_2 _or naturally occurring isotopes [28]. For each scenario a comprehensive data set of ^13^C labeling data (Table 4) and stoichiometric data (Tables 2, 3) was used to calculate the fluxes.

### Metabolic fluxes

The obtained flux distributions for *S. cerevisiae *growing in different physiological states are shown in Figure 3. The fluxes given are relative values normalized to the corresponding specific glucose uptake rate. The examined *S. cerevisiae *strain revealed the typical behavior linked to the switch from respiration to fermentation with increasing growth rate. This involved a redirection of flux from the TCA cycle towards the fermentative pathways. In addition to these previously described, well-known changes, a number of additional pathway fluxes were, however, affected by the change of the physiological regime.

#### PPP and glycolysis

The metabolic shift of *S. cerevisiae *was accompanied by a substantial decrease of the relative flux through the PPP (Figure 3). The decreasing contribution of the PPP with increasing growth rate has direct consequences for the supply of NADPH, required in various anabolic reactions, and certain anabolic precursors. Taking into account that two molecules NADPH are generated in the oxidative part of the PPP, the relative supply of NADPH is 110 % (normalized to the glucose uptake rate) at μ = 0.15 h^-1^. In comparison only 60 % and 30 % NADPH, respectively, are formed at μ = 0.30 h^-1 ^and μ = 0.4 h^-1^. A similar trend can be seen for erythrose 4-phosphate and ribose 5-phosphate. The supply of these anabolic precursors was substantially lower under fermentative conditions.

#### Fluxes around the pyruvate node

Distinct flux changes were observed for the pathways utilizing cytosolic pyruvate, i. e. pyruvate carboxylase, pyruvate decarboxylase, and the pyruvate transporter into the mitochondrion. A surprising result was the increase of flux through pyruvate carboxylase with increasing growth rate. This reaction was thought to mainly serve for the supply of cytosolic oxaloacetate for anabolism. Under fermentative conditions at higher growth rate, however, the anabolic demand for this precursor was much lower as compared to purely oxidative growth (Table 2). Accordingly the increase of flux through pyruvate carboxylase was not driven by an increased anabolic demand. It was in fact linked to increased transport of oxaloacetate into the mitochondrion. Interestingly, pyruvate decarboxylase, the entry step towards the fermentative metabolism, exhibited *in vivo *activity not only during the formation of fermentative by-products. Significant flux through this pathway was also observed under conditions of purely oxidative growth, *i.e. *in the absence of overflow metabolism. Accordingly a high production of acetaldehyde resulted for all physiological states. Under purely oxidative growth, the formed acetaldehyde was completely utilized for the synthesis of cytosolic acetyl CoA. This was then either transported into the mitochondrion or directly channeled into anabolic pathways located in the cytoplasm. During the switch to fermentation the acetaldehyde formed was mainly channeled into the fermentative by-products. The highest flux through pyruvate decarboxylase was observed at maximum production of ethanol and acetate. The ingoing flux through the lower glycolytic chain was higher during fermentative growth as compared to purely oxidative growth (Figure 3).

#### Intercompartmental transport

A substantial transport of carbon from the cytosol into the mitochondrion resulted for all growth rates (Figure 3). Hereby the relative contribution of the different transporters depended on the physiological state. Under purely oxidative growth acetyl CoA, formed in high amounts via the fermentative routes, was the dominating metabolite transported into the mitochondrion. The acetyl CoA transport was strongly decreased under conditions of respiro-fermentative growth. During mainly fermentative metabolism the transport net flux practically decreased to zero. The flux of oxaloacetate transport increased with increasing growth rate. This is linked, as described above, with the increased flux through pyruvate carboxylase. Interestingly the activity of the oxaloacetate transporter correlated with the flux through malic enzyme. This might suggest that sufficient intercompartmental oxaloacetate transport is important for the activity of malic enzyme. The picture observed for the pyruvate transport was more complex. The relative transport flux increased from purely oxidative to respiro-fermentative growth. With further increase of the specific growth rate it substantially decreased. The total flux of carbon into the mitochondrion, as sum of the single transport reactions, was highest during purely oxidative growth.

#### TCA cycle

Overall, the metabolic shift from pure oxidation to fermentation was linked to a strong decrease of the TCA cycle flux (Figure 3). Under purely oxidative growth citrate synthase, the entry enzyme of this pathway, exhibited a relative flux of 73.1 %. The relative activity of this enzyme was 54 % at μ = 0.30 h^-1 ^which indicates that the TCA cycle still contributed to a large extent to the metabolic activity. At μ = 0.40 h^-1 ^the TCA cycle still operated as a cycle, but only at a relatively low activity. The observed flux changes include a shift of the relative contribution of fumarase and the oxaloacetate transporter to the supply of the mitochondrial malate/oxaloacetate pool.

#### Anabolism

Under purely oxidative growth both pathways for glycine biosynthesis were active, whereby the major fraction of glycine was formed via the serine pathway (Figure 3). The simultaneous *in vivo *activity of both pathways was maintained under fermentative conditions. An interesting finding resulted for the biosynthesis of alanine for biomass production. Whereas alanine was exclusively synthesized via the mitochondrial route at purely oxidative growth, cells partly switched to cytosolic alanine formation during the metabolic shift towards fermentation (Figure 3). Under mainly fermentative conditions the cytosolic pathway even provided the major fraction of proteinogenic alanine.

#### Goodness of fit and statistical flux evaluation

For the flux distributions, which are shown in Figure 3, an excellent agreement between experimentally determined and calculated labeling patterns was achieved. The deviation between measured and calculated mass isotopomers was typically below 3 % and thus rather small (Table 4). Generally the intracellular fluxes could be determined with high precision as indicated by the small 90 % confidence intervals (Table 5). This allows an unambiguous differentiation between the different physiological states of the cells. In the present work, the PPP reactions of transaldolase and transketolase were regarded reversible. Comparative flux calculations, setting these reactions as irreversible did lead to a significantly worse fit of the data, which was most pronounced for phenylalanine and tyrosine formed from precursors of the PPP. A model with irreversible PPP reactions thus could not describe the experimental data properly. Moreover this was also related to different results for several flux parameters such as the flux partitioning between glycolysis and PPP. The same was observed for glucose 6-phosphate isomerase. It seems therefore important to fully consider the reversible nature of the PPP reactions in metabolic flux analysis with *S. cerevisiae *as previously proposed by metabolic simulation studies [28,29].

## Discussion

In the present work comparative ^13^C metabolic flux analysis of *S. cerevisiae *at different defined physiological states revealed that the transition from oxidative to fermentative utilization of glucose is reflected by substantial flux changes throughout the whole central carbon metabolism. The different physiological conditions, i. e. purely oxidative, mixed respiro-fermentative and mainly fermentative growth were established by variation of the dilution rate in chemostat culture. Hereby, the extent of the fermentation could be precisely adjusted and run into metabolic and isotopic steady-state.

We observed the previously described transition from respiration to fermentation as indicated by the redirection of flux from the TCA cycle towards the fermentation pathways [8]. It becomes, however, clear from the present study, that the regulatory changes linked to the metabolic transition were not restricted to these reactions, but affected a number of additional pathways.

(i) The metabolic shift had a strong effect on the flux partitioning between glycolysis and PPP. Whereas, at purely oxidative metabolism, the major carbon flux was channeled into the PPP, the opposite was observed for fermentative metabolism. The data of the present work together with previous flux studies of *S. cerevisiae *under various conditions clearly show that the flux partitioning between glycolysis and PPP is growth rate dependent (Figure 4A). A similar relation is found for the flux partitioning and the biomass yield. The reduced biomass yield at higher growth rates results in a reduced demand for erythrose 4-phosphate, ribose 5-phosphate and NADPH, respectively, which are all supplied by the PPP [45]. In the present work a significant amount of carbon was recycled from the PPP into glycolysis via fructose 6-phosphate and glyceraldehyde 3-phosphate by the reversible reactions of the PPP. This indicates that the demand for NADPH, but not for carbon precursors, determined the flux towards the PPP. A detailed discussion of the NADPH metabolism is given below. Previous enzymatic studies of *S. cerevisiae *in chemostat culture revealed that the *in vitro *activity, *i.e. *the capacity, of glucose 6-phosphate dehydrogenase slightly increased with the dilution rate between 0.1 h^-1 ^and 0.4 h^-1 ^[9]. The reduced PPP flux is therefore not due to a lack of capacity of glucose 6-phosphate dehydrogenase. In comparison to other studies the relative PPP flux observed under purely oxidative growth was slightly higher [27]. Due to the fact that this flux parameter could be determined with relatively high precision (Table 5) it seems likely that differences in cultivation conditions or the used strains are responsible for this.

(ii) Pyruvate decarboxylase, the key enzyme of fermentative metabolism, was found to already operate at a high activity under conditions in which alcoholic fermentation was absent (Figure 3). The substantial amount of acetaldehyde formed under these conditions was completely converted into cytosolic acetyl CoA which is either used for anabolism or transported into the mitochondrion. These findings disprove the common perception that only a small fraction of pyruvate is channeled through the fermentative by-pass at low glycolytic flux [30], but rather support previous speculations on fluxes around the pyruvate node linked to the Crabtree effect in *S. cerevisiae *[7]. Based on enzyme profiles, substrate affinities and intracellular pyruvate level studied in chemostat cultivation of *S. cerevisiae *at varied dilution rate it was previously supposed that pyruvate decarboxylase might be active even during purely oxidative metabolism leading to the formation of cytosolic acetyl CoA and its transport into the mitochondrion. The increased pyruvate level observed during fermentative growth [7] could also be an explanation for the observed flux redirection from mitochondrial to cytosolic alanine biosynthesis.

(iii) We found evidence for substantial *in vivo *activity of malic enzyme, as was previously reported for *S. cerevisiae *[29,35]. Additionally we could show a strong flux increase for malic enzyme, when the cells switched from pure oxidation to fermentative metabolism. The flux through malic enzyme increased almost linearly with increasing growth rate (Figure 4C). Although the present work and previous studies differ, concerning *e.g. *the investigated strain, the pH value or the nutrient status, the flux through malic enzyme is generally increased at high growth rate. As exception only a minor flux of 7 % was observed for malic enzyme in a batch culture of *S. cerevisiae *grown on glucose [27]. This might arise from the above mentioned differences in the experimental setup or the generally high uncertainty for the quantification of this flux parameter (Table 5). The exact physiological role of malic enzyme in *S. cerevisiae *so far remained quite intriguing [27]. Also this study does not really provide a satisfying insight into the function of malic enzyme. What at least can be stated is that the flux through malic enzyme seems to be related to the flux through the cytosolic pyruvate carboxylase and the oxaloacetate transporter. One might conclude that the increased activity of malic enzyme results indirectly from the flux redirection at the cytosolic pyruvate node.

(iv) The obtained fluxes allow a detailed inspection of the NADPH metabolism during the metabolic shift. Hereby, it should be noted that the performed flux analysis did not include a balance for NADPH. The fluxes reactions through NADPH supplying reactions were determined only from the fit of the labeling data and the metabolite balances given below. Generally *S. cerevisiae *can recruit different enzymes for the formation of NADPH involving the glucose 6-phosphate dehydrogenase and 6-phosphogluconate dehydrogenase in the PPP [31], NADPH specific acetaldehyde dehydrogenase [32], and NADPH specific isocitrate dehydrogenase [33]. Also malic enzyme is potentially capable to supply NADPH [34]. Clear differences resulted for the different physiological conditions (Figure 5). Linked to the strong decrease of the biomass yield, the anabolic NADPH demand decreased with increasing growth rate. To some extent this is attenuated by the higher protein content of *S. cerevisiae *at high growth rate and the correspondingly increased stoichiometric NADPH demand (Tables 1, 2). We additionally found that sources of NADPH varied with the physiological state.

During purely oxidative growth the PPP alone can account for the total anabolic NADPH demand. Under these conditions an additional NADPH supply by other reactions seems not necessary. Because no intercompartmental transport system for NADPH has been described for *S. cerevisiae*, it is likely that also mitochondrial enzymes are involved in the supply of NADPH. A potential candidate is mitochondrial NADPH specific isocitrate dehydrogenase [33]. This enzyme can, however, account only for a certain fraction of the total flux through the TCA cycle. An exclusive activity of NADPH specific isocitrate dehydrogenase can be excluded. Assuming this the total amount of NADPH supplied would be almost twice as high as the anabolic demand. Thus we conclude that to large extent mitochondrial NADH specific isoenzymes of isocitrate dehydrogenase contribute to the TCA cycle flux during oxidative growth of *S. cerevisiae*.

Under predominantly fermentative utilization of glucose only about 60 % of the totally required NADPH was provided by the PPP. This is an indication for an additional cytosolic NADPH source required during fermentative metabolism in *S. cerevisiae*. *S. cerevisiae *possesses several isoenzymes for acetaldehyde dehydrogenase with different specificity for NADH and NADPH [35]. Possibly the relative contribution of the different isoenzymes changes with the on-set of fermentation, so that instead of mainly NADH under oxidative growth, also NADPH is formed. Recently it was found that especially the NADPH dependent acetaldehyde dehydrogenase isoforms are involved in anaerobic fermentation of *S. cerevisiae *[35]. The increased NADPH formation by acetaldehyde dehydrogenase would probably lead to an increased level of intracellular NADPH, known to inhibit the PPP enzymes glucose 6-phosphate dehydrogenase and 6-phosphogluconate dehydrogenase [31]. The reduction of the PPP flux might therefore not only be caused by the reduced anabolic demand, but also by the activation of alternative cytosolic pathways for NADPH supply.

**(v) **An insight into the limiting reactions, involved in the complex flux redirection during the metabolic shift, can be derived from absolute carbon fluxes. As can be calculated from the relative fluxes and the specific glucose uptake rate (Figure 3) the transition from oxidative to fermentative metabolism in *S. cerevisiae *was accompanied by a more than 5-fold increase of the specific glucose uptake flux. It is interesting to see, how the increased influx of carbon is distributed throughout the metabolic network. The absolute carbon flux of enzymes involved in fermentation and the transport of cytosolic acetyl CoA into the mitochondrion are shown in Figure 6A. Pyruvate decarboxylase exhibits a tremendous flux increase with increasing growth rate. A completely different picture results for the enzymes catalyzing the subsequent metabolic steps, *i.e. *acetaldehyde dehydrogenase, acetyl CoA synthase and the transporter. The absolute carbon flux through acetyl CoA synthase remained constant for all physiological states investigated. Obviously this enzyme was already working at maximum capacity during purely oxidative growth. The saturation of acetyl CoA synthase obviously initiated the secretion of acetate into the medium with increasing growth rate. Additionally a strong decrease of the intracellular acetyl CoA level appears very likely. The insufficient activity of this enzyme had also consequences for the intercompartmental acetyl CoA transport. As shown, the absolute carbon flux through the transporter decreased to practically zero (Figure 6A). The cytosolic acetyl CoA was in fact completely channeled into anabolic reactions. Probably the reactions withdrawing cytosolic acetyl CoA into anabolism are favored by a higher affinity of the catalyzing enzymes. The insufficient capacity of acetaldehyde dehydrogenase and acetyl CoA synthase could even limit the supply of cytosolic acetyl CoA for anabolism, *i.e. *the formation of lysine and lipids. In this regard a significant decrease of the *in vitro *activity, i. e. the capacity, of acetaldehyde dehydrogenase and acetyl CoA synthase was previously observed during the metabolic shift from oxidative to fermentative metabolism [9]. It was concluded that probably these enzymes display the limiting steps that cause the secretion of ethanol and acetate at high growth rate. The present study provides further evidence that indeed these reactions are key points with respect to increased production of ethanol and acetate. The same principle can be applied to study the reactions downstream of the pyruvate pool (Figure 6B). The absolute carbon flux of pyruvate dehydrogenase was low under purely oxidative growth, but showed no further increase towards higher growth rate, despite the carbon flux entering the cytosolic pyruvate pool, and thus potentially available, was much higher. This indicates that pyruvate dehydrogenase already operates at maximum capacity during respiro-fermentative growth at μ = 0.3 h^-1^. The excess carbon entering the cytosolic pyruvate pool is obviously directed towards pyruvate carboxylase and fermentation. Probably also the activation of cytosolic alanine synthesis is related to this phenomenon. Pyruvate dehydrogenase thus displays an additional bottleneck. The insufficient capacity of pyruvate dehydrogenase seems to have an influence on the transport of pyruvate into the mitochondrion. Due to the fact that, during fermentative growth, a substantial fraction of mitochondrial pyruvate is already supplied via malic enzyme, the absolute carbon flux of the pyruvate transport is lower (Figure 6B). The absolute carbon flux through glucose 6-phosphate dehydrogenase increased about 1.5-fold when the dilution rate was increased from 0.15 h^-1 ^to 0.4 h^-1^. In a previous study it was shown that, linked to the same shift in dilution rate, the intracellular level of this enzyme was increased about 1.6-fold [9]. Obviously, both glucose 6-phosphate dehydrogenase level and NADPH level are involved in the regulation of carbon flux into the PPP. In case of acetaldehyde dehydrogenase and acetyl CoA synthase the limited carbon flux at high dilution rate seems to be related to a decrease in enzyme level [9]. Additionally inhibitory effects, *e.g. *of acetaldehyde on acetaldehyde dehydrogenase [9], might be involved. The present study suggests that the flux redirection, caused by the regulatory network linked to the Crabtree effect, leads to a saturation of the capacity, *i.e. *the absolute flux, of acetaldehyde dehydrogenase, acetyl CoA synthase, and pyruvate dehydrogenase. It is the limitation of these enzymes that is then mainly responsible for the onset of the production of fermentative by-products during growth of *S. cerevisiae *at high growth rate.

## Conclusion

Summarizing, the regulatory changes linked to the metabolic transition affect a number of pathways throughout the whole central metabolism of *S. cerevisiae*. It is known that yeast undergoes various changes involving *e. g. *change of enzyme levels, tolerance to stress, or morphology, when the metabolism switches from respirative to fermentative growth. These changes are controlled complex regulatory systems of transcriptional control as well as post-transcriptional control [36]. One of the important primary signals for the regulation of yeast metabolism under different nutrient conditions is the concentration of glucose [37]. As shown the glucose concentration in the medium indeed increased with increasing growth rate (Figure 2). The on-set of the glucose repression system was probably the major signal that triggered the observed changes in the intracellular carbon fluxes. This includes the observed decrease of flux through the TCA cycle with increasing growth rate, which probably results from direct repression of TCA cycle genes on the transcriptional level by the glucose repression system [38]. Similarly the strong increase of the specific glucose uptake rate and the glycolytic flux is probably a direct result of transcriptional induction of the corresponding genes [39]. Recent studies on fluxes and transcriptional levels revealed that a number of intracellular pathways in *S. cerevisiae *is probably regulated on the post-transcriptional level [36]. Metabolic flux analysis alone on itself as performed in the present study cannot distinguish between flux changes directly controlled or indirectly affected *e.g. *by regulation of interconnected reactions. However, it appears as a valuable tool to quantitatively assess metabolic changes in great detail and to provide important data leading to a deeper understanding of the metabolism of *S. cerevisiae*.

## Methods

### Organism and cultivation

*S. cerevisiae *ATCC 32167 was purchased from the American Type Strain and Culture Collection (Manassas, USA). The mineral medium used for cultivation contained 2 g/L glucose, 0.5 g/L (NH_4_)_2_HPO_4_, 1.0 g/L (NH_4_)_2_SO_4_, 0.05 g/L MgSO_4 _·7 H_2_O, 0.025 g/L citric acid, 0.5 g/L KCl, 0.03 g/L CaCl_2_·2 H_2_O, 3 mg/L FeCl_3_·6 H_2_O, 2.1 mg/L MnSO_4_·H_2_O, 1.8 mg/L ZnSO_4_·7 H_2_O, 0.5 mg/L CuSO_4_·5 H_2_O, 60.3 mg/L *myo*-inositol, 30 mg/L Ca-panthotenate, 6 mg/L thiamin·HCl, 1.5 mg/L pyridoxine·HCl, 0.03 mg/L biotin, and 50 mmol/L phosphate buffer (pH 6.2). All chemicals were of analytical grade and purchased from Fluka (Buchs, Switzerland), Merck (Darmstadt, Germany), or Sigma (St. Louis, USA). In tracer studies normal glucose was replaced by an equal molar concentration of 99% [1-^13^C] glucose (Campro, Veenendal, Netherlands). Cultivations were performed in a continuously operated bioreactor (Meredos, Bovenden, Germany) with a culture volume of 100 ml at 30°C and 500 rpm. The aeration rate was kept at 100 mL/min by a mass flow controller (WMR Compact 4, Brooks Instruments, Veenendal, Netherlands). Composition of inlet and exhaust gas was determined on-line by mass spectrometry as described previously [40]. For flux quantification the culture was operated with the tracer substrate for five residence times, including on-line verification by a constant CO_2 _concentration in the exhaust gas, so that metabolic and isotopic steady-state could be assumed. Samples were then taken continuously from the outlet of the reactor and harvested at 4°C. Supernatant, obtained by centrifugation (16000 g, 5 min, Biofuge Pico, Heraeus, Hanau, Germany), was used for analysis of extracellular metabolites. The cell pellet was used for ^13^C labeling analysis by GC-MS.

### Analysis of biomass and extracellular metabolites

Optical density (OD_660_) was measured at 660 nm (Novaspec II, Pharmacia, Freiburg, Germany). Cell dry mass (CDM) was determined gravimetrically after two-fold washing, centrifugation (10 min, 4°C, 3940 g; Labofuge 400 R, Heraeus, Hanau, Germany), and drying at 80°C until constant weight. A linear relationship of OD_660 _= 0.52 [g CDM /L] was obtained. Enzymatic test-kits were used for quantification of glucose, glycerol, acetate (Boehringer Mannheim, Mannheim, Germany) and ethanol (Sigma Diagnostics, St. Louis, USA) in the supernatant. Concentrations of 2-oxoglutarate, pyruvate, succinate, and fumarate in the supernatant were analyzed by HPLC [40]. Amino acid composition of the cell protein of *S. cerevisiae *was quantified by HPLC [41] after hydrolysis with 6 M HCl for 24 h at 105°C. The mean relative error of the stoichiometric measurements, *i.e. *the quantification of the yield coefficients, was 3.7 % (biomass yield), 4.0 % (ethanol yield), 9.3 % (acetate yield) and 11.3 % (glycerol yield).

### GC-MS analysis of proteinogenic amino acids

Measurement of amino acid labeling patterns from cell hydrolysate was carried out by GC-MS in selective ion monitoring mode [42]. For a number of ion clusters all mass isotopomer fractions from the non-labeled (x_0_) to the fully ^13^C labeled (x_n_) variant could be measured with good signal intensity and accuracy (Table 4). Accordingly, a comprehensive data set was available for calculation of the metabolic fluxes. The relative measurement error for a mass isotopomer fraction from the four replicates at each dilution rate was typically below 1 %. It must be mentioned that some amino acids could not be considered for the flux calculation. These were tryptophan, methionine, cysteine and histidine, which did not yield suitable GC/MS signals, proline, which exhibited isobaric overlay, and leucine and isoleucine with ambiguous fragmentation pathways due to similarity of the amino acid side chain and the derivatization agent.

### Metabolic network of *S. cerevisiae*

**Figure 1:**
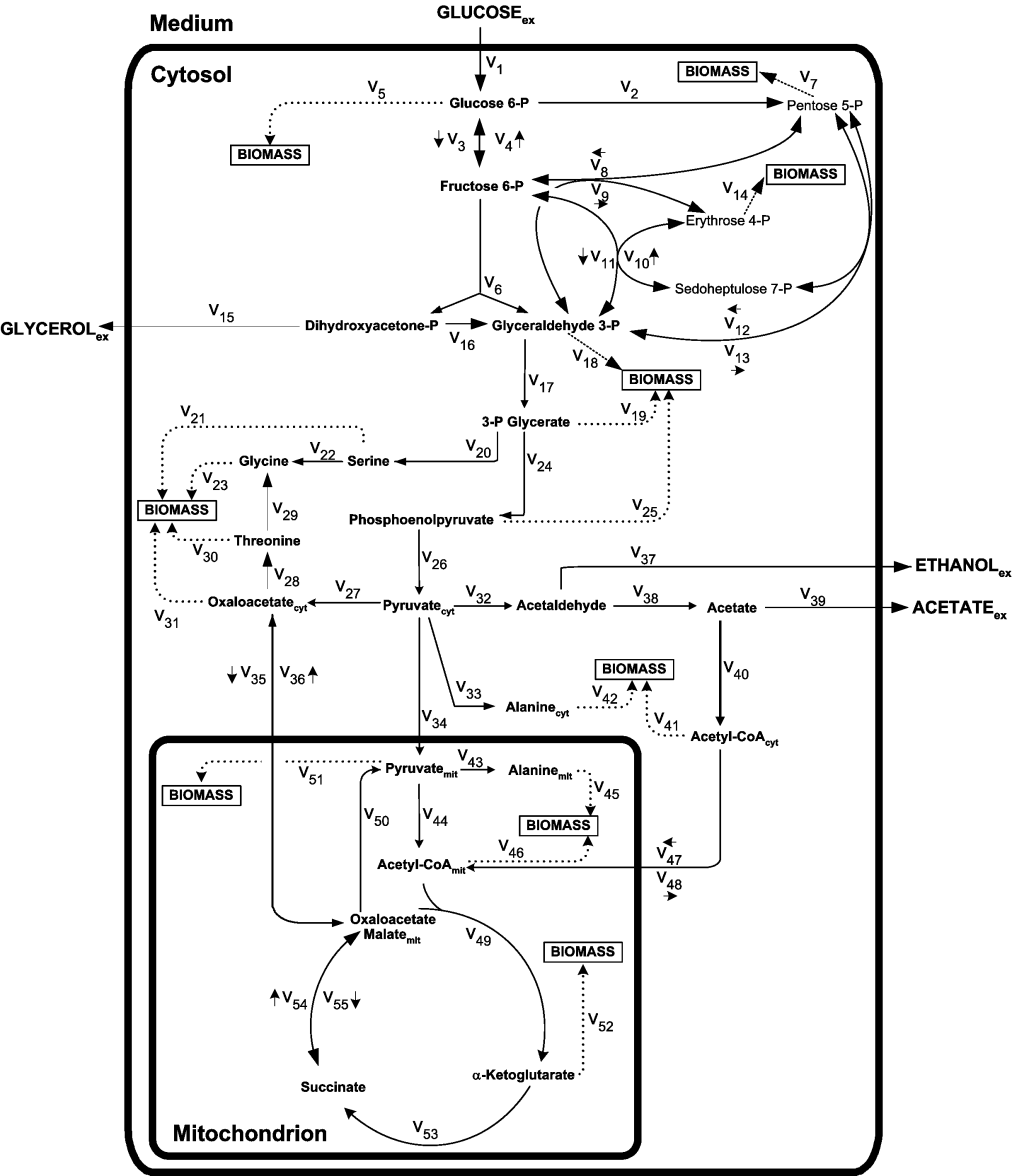
Metabolic network of the central cytosolic and mitochondrial metabolism of *S. cerevisiae *investigated in the present work. The network comprises glycolysis, pentose phosphate pathway, anaplerotic carboxylation, fermentative pathways, inter-compartmental transport of acetyl-CoA, pyruvate, and oxaloacetate, respectively, TCA cycle, malic enzyme and anabolic reactions from intermediary metabolites into anabolism.

**Figure 2:**
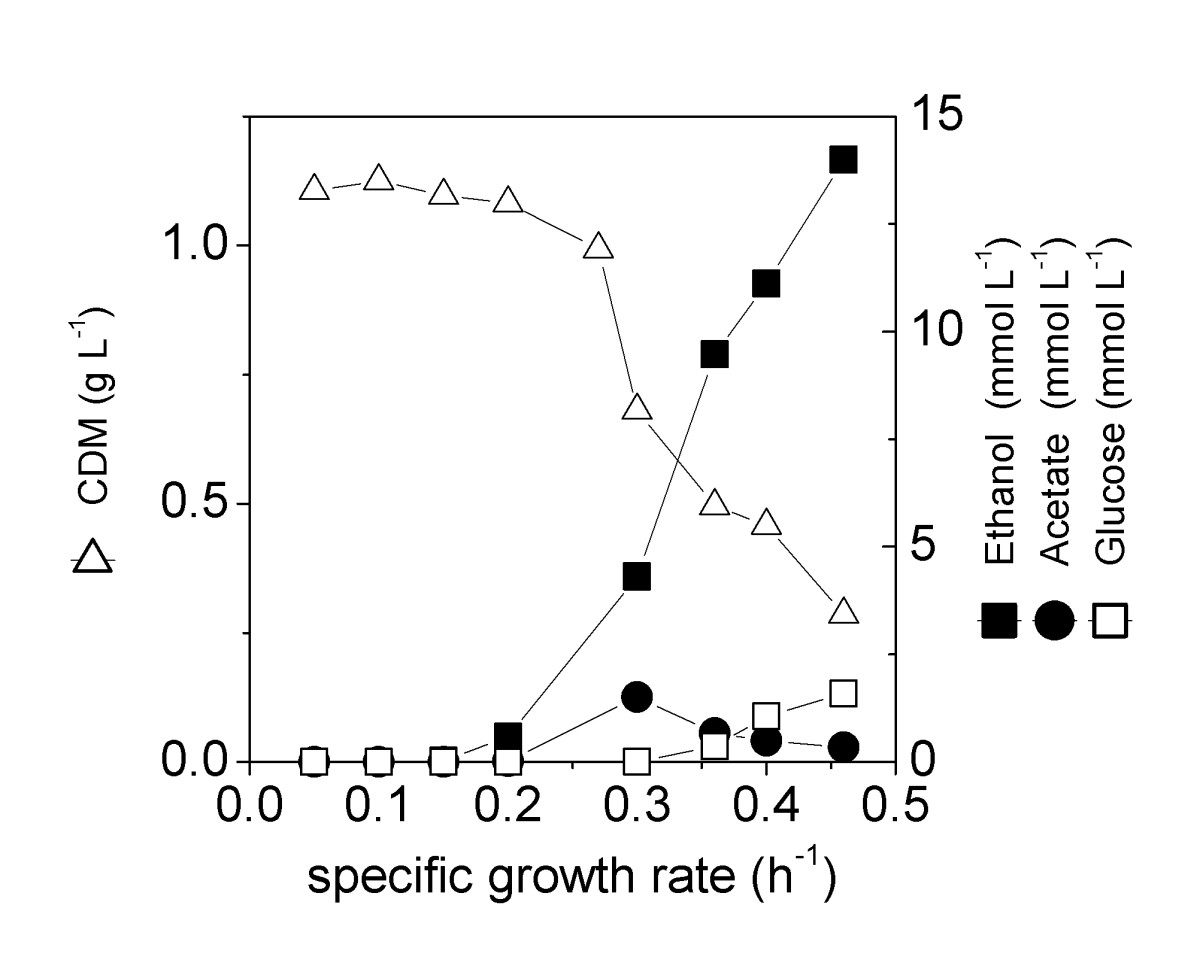
Cultivation profile of *S. cerevisiae *in chemostat under aerobic, glucose-limited conditions at different growth rates: Concentration of cell dry mass (CDM), ethanol, acetate, and glucose.

**Figure 3:**
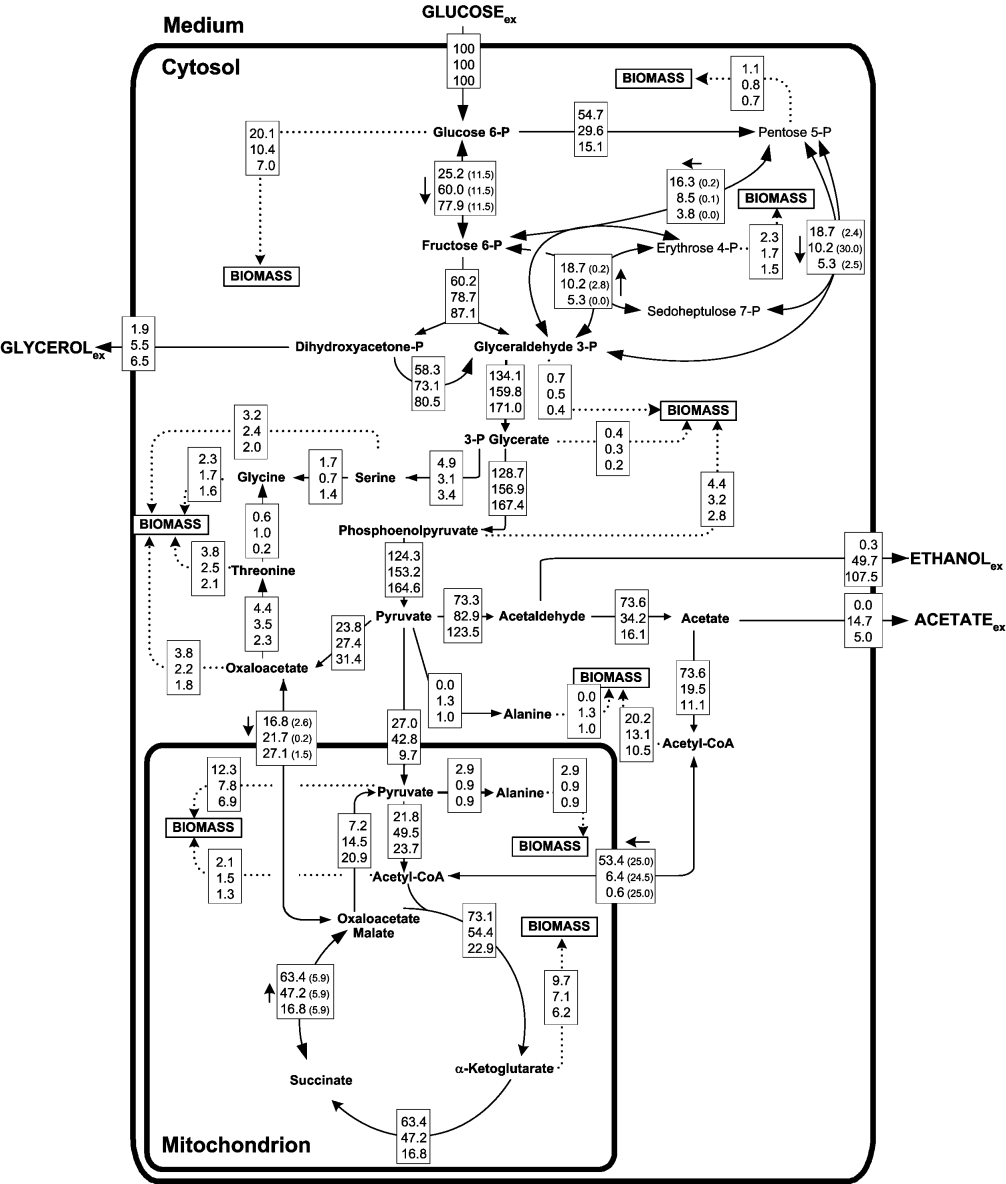
Intracellular carbon flux distribution of *S. cerevisiae *cultivated in chemostat on [1-^13^C] glucose under aerobic glucose-limited conditions at different growth rates. All fluxes are given as relative fluxes normalized to the specific glucose uptake rate. For each reaction the fluxes corresponding to purely oxidative (μ = 0.15 h^-1^, q_glc _= 1.56 mmol g^-1 ^h^-1^), respiro-fermentative (μ = 0.30 h^-1^, q_glc _= 4.90 mmol g^-1 ^h^-1^), and mainly fermentative growth (μ = 0.40 h^-1^, q_glc _= 8.23 mmol g^-1 ^h^-1^), respectively, are shown from top to bottom. For reversible reactions an additional arrow indicates the direction of the net flux and the values in the squared brackets are the obtained reversibilities of the corresponding enzymes. The fluxes correspond to the optimal fit between experimentally determined steady-state ^13^C labeling patterns of amino acids of the cell protein and ^13^C labeling patterns simulated via isotopomer modelling.

**Figure 4:**
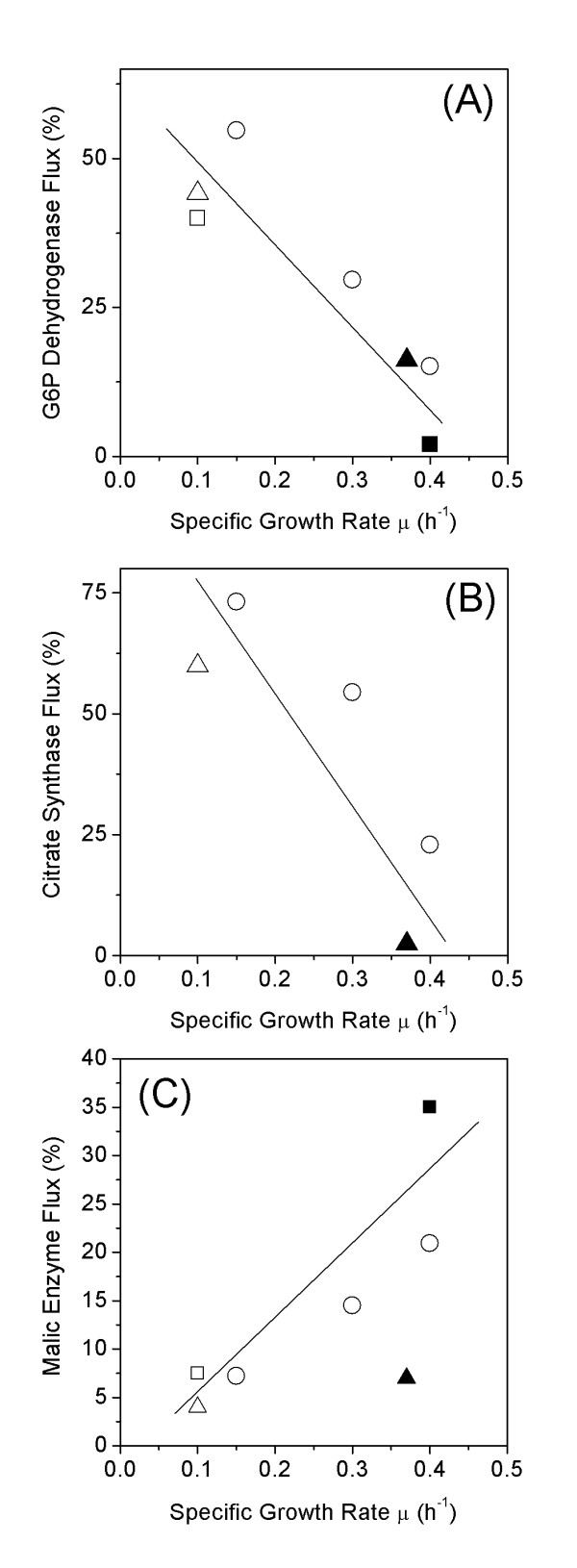
Correlation of specific growth rate and relative carbon fluxes in the central metabolism of *S. cerevisiae*: glucose 6-phosphate dehydrogenase (A), citrate synthase (B), and malic enzyme (C). Data shown comprise fluxes of *S. cerevisiae *ATCC 31267 determined at different growth rates in glucose-limited chemostat (this work, open circle), of *S. cerevisiae *CEN.PK 113.7D at μ = 0.1 h^-1 ^in glucose-limited chemostat ([27],open triangle), at μ = 0.37 h^-1 ^in batch culture under glucose excess ([27], closed triangle), of *S. cerevisiae *CEN.PK 113.7D at μ = 0.1 h^-1 ^in glucose-limited chemostat ([26], open square), at μ = 0.4 h^-1 ^in batch culture under glucose excess ([26], closed square). Note that Fiaux et al. [26] determined flux intervals instead of exact values, whereby the values given here represent the corresponding average of each interval.

**Figure 5:**
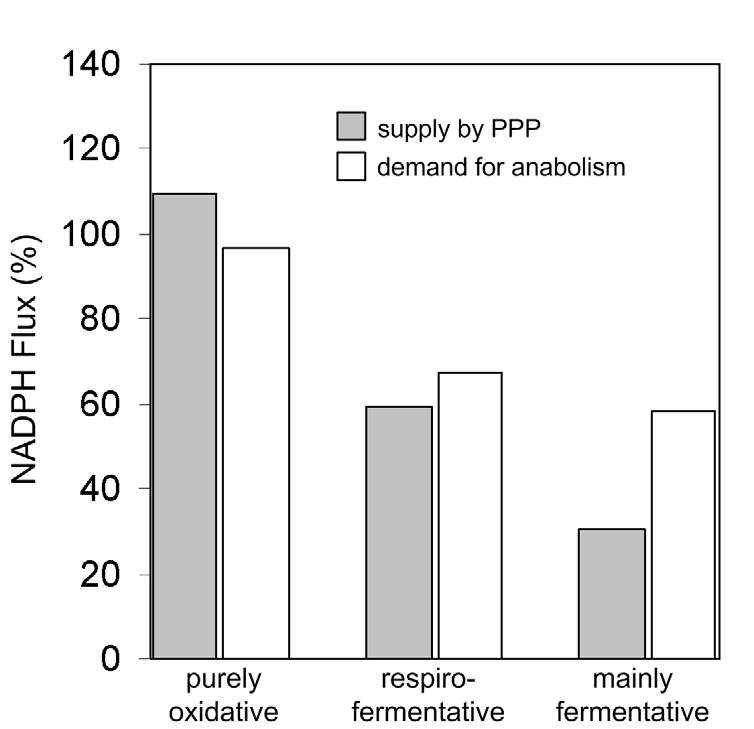
NADPH metabolism of *S. cerevisiae *cultivated in chemostat under aerobic glucose-limited conditions at different growth rates. The fluxes correspond to purely oxidative (μ = 0.15 h^-1^), respiro-fermentative (μ = 0.30 h^-1^), and mainly fermentative growth (μ = 0.40 h^-1^).

**Figure 6:**
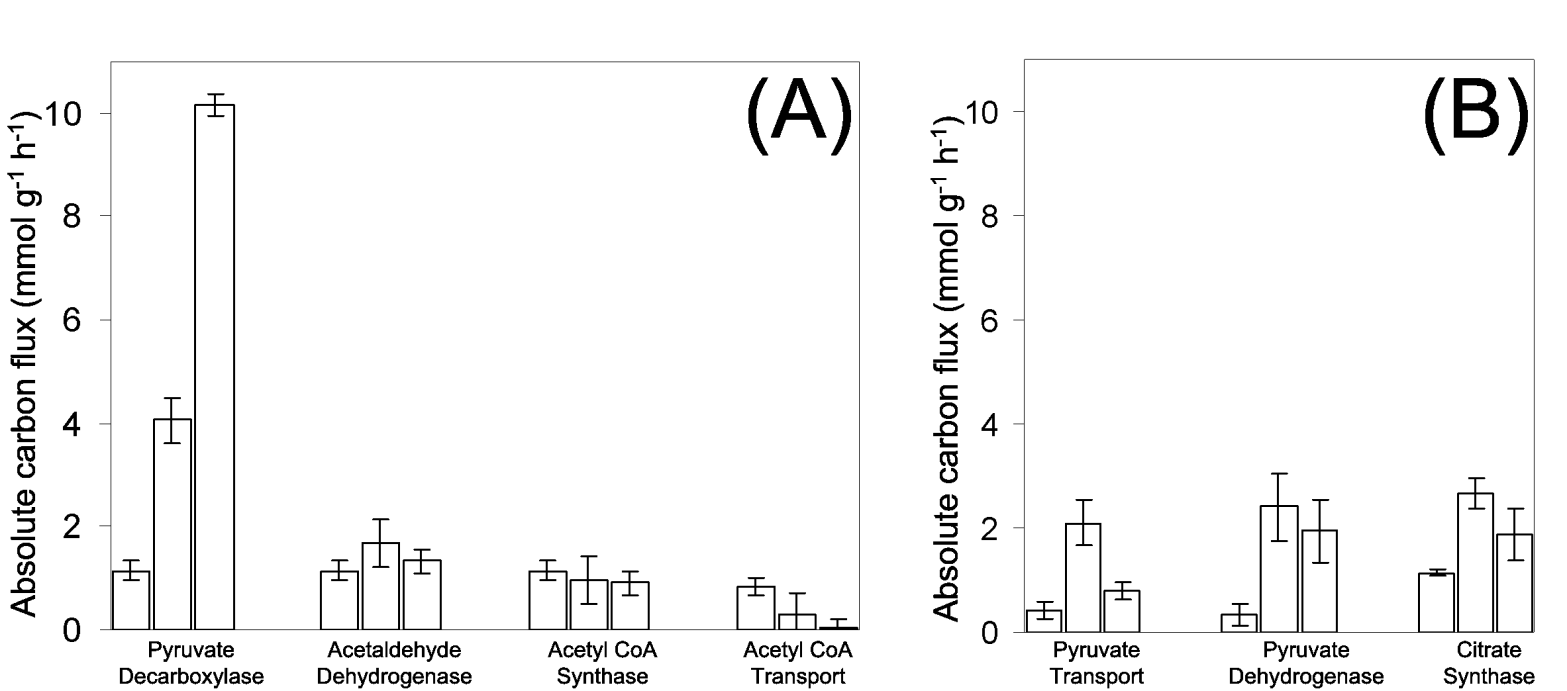
Absolute carbon fluxes of *S. cerevisiae *cultivated in chemostat under aerobic glucose-limited conditions at different growth rates: Fluxes of pyruvate decarboxylase, acetaldehyde dehydrogenase and acetyl CoA synthase (A), and of intercompartmental pyruvate transport, pyruvate dehydrogenase and citrate synthase (B). For each enzyme the flux at purely oxidative (μ = 0.15 h^-1^), respiro-fermentative (μ = 0.30 h^-1^), and mainly fermentative growth (μ = 0.40 h^-1^) is shown from left to right. Additionally the 90 % confidence intervals for the fluxes are given.

The metabolic network of *S. cerevisiae *comprising the major routes of the central carbon metabolism (PPP, glycolysis, fermentative pathways, TCA cycle, and anaplerosis) is shown in Figure 1. As shown, cytosol and mitochondrion are regarded as separate compartments. The model comprises separate pools for pyruvate, oxaloacetate and acetyl CoA in each of the two compartments, respectively. Anaplerotic carboxylation of pyruvate is located in the cytosol [43]. The malic enzyme is considered as mitochondrial reaction [34]. Additionally transport reactions for pyruvate, oxaloacetate and acetyl CoA are considered for intercompartmental carbon exchange [21,27,44]. The transport of pyruvate was assumed to be unidirectional due to its coupling to the proton motive force, whereas the transport reactions for oxaloacetate and acetyl CoA were considered reversible [26]. The reaction catalyzed by glucose 6-phosphate isomerase and the TCA cycle reactions interconverting oxaloacetate and fumarate were regarded as reversible steps, whereby the reversibility of these reactions was taken from the literature [27]. In addition to the central metabolic routes, biosynthetic pathways towards the different amino acids are implemented. In most of the cases amino acid formation is carried out by a single pathway in either one of the two compartments. Exceptions are the biosynthesis of alanine and glycine. For alanine biosynthesis a cytosolic and a mitochondrial pathway was implemented in the model. In addition to the known mitochondrial route, a cytoplasmic alanine amino transferase, generating alanine from cytosolic pyruvate, was recently identified in *S. cerevisiae *[25]. For glycine the alternative route via threonine aldolase [45,46] was taken into account in addition to the synthesis from serine. The following balance equations of intracellular metabolites were formulated for the examined network using the numbering of the fluxes of Figure 1.

### Cytosolic Metabolite Pools

Glucose 6-phosphate: ν_1 _- ν_2 _- ν_3 _+ ν_4 _- ν_5 _= 0     (1)

Fructose 6-phosphate: ν_3 _- ν_4 _- ν_6 _+ ν_8 _- ν_9 _+ ν_10 _- ν_11 _= 0     (2)

Pentose phosphate: ν_2 _- ν_7 _- ν_8 _+ ν_9 _- 2ν_12 _+ 2ν_13 _= 0     (3)

Erythrose 4-phosphate: - ν_8 _+ ν_9 _+ ν_10 _- ν_11 _- ν_14 _= 0     (4)

Sedoheptulose 7-phosphate: ν_12 _- ν_13 _- ν_10 _+ ν_11 _- ν_5 _= 0     (5)

Glyceraldehyde 3-phosphate: ν_6 _- ν_10 _+ ν_11 _+ ν_8 _- ν_9 _+ ν_12 _- ν_13 _+ ν_16 _- ν_17 _- ν_18 _= 0     (6)

Dihydroxyacetone-phosphate: ν_6 _- ν_15 _- ν_16 _= 0     (7)

3-Phosphoglycerate: ν_17 _- ν_19 _- ν_20 _- ν_24 _= 0     (8)

Serine: ν_20 _- ν_21 _- ν_22 _= 0     (9)

Glycine: ν_22 _- ν_23 _+ ν_29 _= 0     (10)

Threonine: ν_28 _- ν_29 _- ν_30 _= 0     (11)

Phosphoenolpyruvate: ν_24 _- ν_25 _- ν_26 _= 0     (12)

Pyruvate_cyt_: ν_26 _- ν_27 _- ν_32 _- ν_33 _- ν_34 _= 0     (13)

Acetaldehyde: ν_32 _- ν_37 _- ν_38 _= 0     (14)

Acetate: ν_38 _- ν_39 _- ν_40 _= 0     (15)

Acetyl CoA_cyt_: ν_40 _- ν_41 _- ν_47 _+ ν_48 _= 0     (16)

Alanine_cyt_: ν_33 _- ν_42 _= 0     (17)

Oxaloacetate_cyt_: ν_27 _- ν_28 _- ν_31 _- ν_35 _+ ν_36 _= 0     (18)

### Mitochondrial Metabolite Pools

Pyruvate_mit_: ν_34 _- ν_43 _- ν_44 _+ ν_50 _- ν_51 _= 0     (19)

Alanine_mit_: ν_43 _- ν_45 _= 0     (20)

Acetyl CoA_mit_: ν_44 _- ν_46 _+ ν_47 _- ν_48 _- ν_49 _= 0     (21)

2-Oxoglutarate: ν_49 _- ν_52 _- ν_53 _= 0     (22)

Succinate: ν_53 _- ν_54 _+ ν_55 _= 0     (23)

Oxaloacetate/Malate_mit_: ν_35 _- ν_36 _- ν_49 _- ν_50 _+ ν_54 _- ν_55 _= 0     (24)

For each examined physiological state a different, growth rate dependent, cellular composition of *S. cerevisiae *was considered (Table 1). The macromolecular composition of a yeast cell in relation to the growth rate was calculated from literature data [47-49]. The composition of each macromolecule, however, was assumed to be identical for the different growth conditions [27]. The content and composition of lipids, nucleic acids, and carbohydrates was taken from the literature [48,49]. The amino acid composition of the cell protein was experimentally determined in this work. Based on these data and the stoichiometry of the anabolic pathways in yeast the demand for the different precursor metabolites could be calculated (Table 2) and used as measured fluxes in the flux estimation routine as described previously [41].

### Mathematical modeling and flux analysis

Flux calculations were carried out with Matlab 6.1 and Simulink 3.0 (Mathworks Inc., Nattick, USA) combining metabolite balancing and isotopomer modeling. The performed approach utilized metabolite balancing (see mass balances given above) during each optimization step considering stoichiometric data on anabolic demand for biomass precursors (Table 2) and product secretion (Table 3). The set of fluxes that gave minimum deviation between experimental and simulated mass isotopomer fractions was taken as best estimate for the intracellular flux distribution. As error criterion a weighted sum of least squares was used [41]. Since the non-linear structure of isotopomer models may potentially lead to local minima, multiple parameter initializations were used to assure that the obtained flux distributions represented a global optimum. Statistical analysis of the obtained flux parameters was carried out by Monte-Carlo analysis, whereby random, normal-distributed noise was added to the measurement data [41,50].

## Authors' contributions

OF carried out the experimental work including the chemostat cultivations, the ^13^C tracer studies and all analytics involved.

CW designed the study and carried out all computational work involving the construction of the metabolic network model of *S. cerevisiae*, the software implementation, the calculation of carbon fluxes, and the statistical analysis. He drafted and composed the manuscript.

Both authors read and approved the final manuscript.

## Figures and Tables

**Table 1:** Macromolecular composition of *Saccharomyces cerevisiae *in glucose-limited chemostat culture at different dilution rates. The data given in g (g cell dry mass)^-1 ^are calculated from literature data [27, 47-49].

Compound	D = μ = 0.15 h^-1^	D = μ = 0.30 h^-1^	D = μ = 0.40 h^-1^
Protein	0.415	0.480	0.524
Carbohydrate	0.372	0.301	0.254
Lipids	0.070	0.070	0.070
RNA+DNA	0.069	0.096	0.113

**Table 2:** Anabolic demand of *Saccharomyces cerevisiae *during aerobic glucose-limited chemostat culture at different dilution rates. The data are given in μmol (g cell dry mass)^-1 ^and are calculated from the cellular composition.

Compound	D = μ = 0.15 h^-1^	D = μ = 0.30 h^-1^	D = μ = 0.40 h^-1^
Glucose 6-phosphate	2089	1694	1431
Erythrose 4-phosphate	243	281	307
Ribose 5-phosphate	115	127	141
Glyceraldehyde 3-phosphate	77	77	77
Phosphoglycerate (for lipids, nucleotides)	44	44	44
Phosphoglycerate (for serine + cysteine)	343	397	433
Phosphoglycerate/Oxaloacetate (for glycine)^a^	236	273	298
Oxaloacetate (for threonine, methionine, isoleucine)	393	455	496
Oxaloacetate (for others)	290	332	364
Phosphoenolpyruvate	457	529	577
Pyruvate (for alanine)^b^	304	352	384
Pyruvate (mitochondrial, for others)	981	1135	1237
Acetyl-CoA (cytosolic)	2108	2142	2164
Acetyl-CoA (mitochondrial)	216	250	273
2-Oxoglutarate	1008	1166	1272
NADPH	10088	11206	11956

^a ^The actual precursor demand for glycine biosynthesis depends on the contribution of the serine pathway (from phosphoglycerate) and threonine aldolase (from oxaloacetate). Based on the relative activity of both pathways, the corresponding demand has to be added appropriately to phosphoglycerate and oxaloacetate, respectively.

^b ^The demand of cytosolic and mitochondrial pyruvate for alanine synthesis depends on the relative contribution of cytosolic and mitochondrial route to alanine synthesis.

**Table 3:** Specific growth, substrate consumption, product formation rates and carbon balance of *Saccharomyces cerevisiae *cultured on [1-^13^C] glucose in chemostat at different growth rates for metabolic flux analysis.

**Specific rate (C mmol h^-1^)**	**Purely Oxidative Growth (μ = 0.15 h^-1^)**	**Respiro-Fermentative Growth (μ = 0.30 h^-1^)**	**Mainly Fermentative Growth (μ = 0.40 h^-1^)**
Glucose	- 0.96	- 1.88	- 2.30
Organic acids	0.01	0.01	0.01
Ethanol	0.00	0.31	0.82
Glycerol	0.01	0.05	0.08
Acetate	0.00	0.09	0.04
CDM	0.55	0.69	0.67
CO_2_	0.37	0.66	0.64
sum of substrates	- 0.96	- 1.88	- 2.30
sum of products	0.94	1.81	2.26
carbon recovery [%]	**97.9**	**96.3**	**98.3**

**Table 4:** ^13^C labeling patterns of amino acids in cell hydrolysates of *Saccharomyces cerevisiae *cultivatedon [1-^13^C] glucose in chemostat under aerobic glucose-limited conditions at different growth rates.

**Measured Metabolite**	**Purely Oxidative Growth **(μ = 0.15 h^-1^)			**Respiro-Fermentative Growth **(μ = 0.30 h^-1^)			**Mainly Fermentative Growth **(μ = 0.40 h^-1^)		
	M_0_	M_1_	M_2_	M_0_	M_1_	M_2_	M_0_	M_1_	M_2_
Alanine (*m/z *260, C_1–3_)									
calc^a^	0.647	0.328		0.596	0.380		0.539	0.435	
exp^b^	0.652	0.331		0.597	0.381		0.543	0.442	
									
Alanine (*m/z *232, C_2–3_)									
calc	0.671	0.316		0.618	0.372		0.561	0.427	
exp	0.666	0.325		0.615	0.374		0.549	0.442	
									
Glycine (*m/z *246, C_2_)									
calc	0.922	0.074		0.932	0.065		0.964	0.035	
exp	0.922	0.074		0.931	0.064		0.963	0.036	
									
Valine (*m/z *186, C_2–5_)									
calc	0.450	0.424	0.117	0.365	0.457	0.165	0.308	0.471	0.203
exp	0.442	0.424	0.116	0.361	0.451	0.165	0.301	0.480	0.203
									
Valine (*m/z *260, C_2–5_)									
calc	0.450	0.424	0.117	0.365	0.457	0.165	0.308	0.471	0.203
exp	0.442	0.428	0.120	0.364	0.457	0.170	0.300	0.475	0.206
									
Valine (*m/z *288, C_2–5_)									
calc	0.434	0.425	0.128	0.346	0.451	0.179	0.288	0.464	0.220
exp	0.435	0.429	0.125	0.355	0.456	0.174	0.294	0.475	0.210
									
Serine (*m/z *288, C_2–3_)									
calc	0.694	0.303		0.628	0.369		0.566	0.430	
exp	0.693	0.300		0.624	0.366		0.569	0.422	
									
Serine (*m/z *362, C_2–3_)									
calc	0.694	0.303		0.628	0.369		0.566	0.430	
exp	0.693	0.302		0.623	0.369		0.567	0.424	
									
Serine (*m/z *390, C_1–3_)									
calc	0.682	0.311		0.611	0.375		0.560	0.431	
exp	0.682	0.308		0.617	0.370		0.564	0.428	
									
Threonine (*m/z *376, C_2–4_)									
calc	0.541	0.381		0.526	0.409		0.515	0.440	
exp	0.540	0.387		0.521	0.409		0.504	0.455	
									
Threonine (*m/z *404, C_1–4_)									
calc	0.496	0.396		0.504	0.413		0.488	0.444	
exp	0.500	0.398		0.505	0.411		0.496	0.455	
									
Aspartate (*m/z *418, C_1–4_)									
calc	0.496	0.396		0.504	0.413		0.488	0.444	
exp	0.502	0.399		0.506	0.415		0.499	0.454	
									
Arginine (*m/z *414, C_1–5_)									
calc	0.302	0.417	0.219	0.239	0.410	0.261	0.263	0.450	0.241
exp	0.309	0.415	0.218	0.245	0.406	0.260	0.263	0.450	0.244
									
Phenylalanine (*m/z *336, C_1–9_)									
calc	0.379	0.429	0.166	0.304	0.437	0.212	0.240	0.442	0.262
exp	0.371	0.417	0.166	0.305	0.431	0.212	0.238	0.437	0.263
									
Phenylalanine (*m/z *302, C_1–2_)									
calc	0.973			0.961			0.979		
exp	0.957			0.952			0.975		
									
Tyrosine (*m/z *466, C_1–9_)									
calc	0.379	0.429	0.166	0.304	0.437	0.212	0.240	0.442	0.262
exp	0.379	0.421	0.165	0.304	0.432	0.213	0.242	0.440	0.250
									
Tyrosine (*m/z *302, C_1–2_)									
calc	0.973			0.961			0.979		
exp	0.972			0.964			0.966		
									
Glutamate (*m/z *432, C_1–5_)									
calc	0.352	0.427	0.184	0.278	0.431	0.233	0.276	0.458	0.230
exp	0.353	0.429	0.184	0.278	0.432	0.233	0.279	0.467	0.228

^a ^Calculated values predicted by the solution of the mathematical model corresponding to the optimized set of fluxes

^b ^Experimental values obtained by GC-MS analysis of TBDMS-derivatized amino acids

**Table 5:** Statistical evaluation of metabolic fluxes of *Saccharomyces cerevisiae *cultured in chemostat under aerobic, glucose-limited conditions at μ = 0.15 h^-1^, μ = 0.30 h^-1^, and μ = 0.40 h ^-1^.

Flux Parameter	Interval for 90 % Confidence^a ^(μ = 0.15 h^-1^)	Interval for 90 % Confidence (μ = 0.30 h^-1^)	Interval for 90 % Confidence (μ = 0.40 h^-1^)
**Net Flux**			
glucose 6-phosphate isomerase	[24.7 26.1]	[58.5 61.3]	[77.3 78.9]
fructose 1,6-bisphosphate aldolase	[59.5 61.0]	[77.6 79.6]	[86.3 88.0]
triosephosphate isomerase	[57.6 59.1]	[72.1 74.1]	[79.6 81.6]
glyceraldehyde 3-phosphate dehydrogenase	[132.5 135.9]	[157.7 162.0]	[169.2 173.2]
enolase	[126.9 130.7]	[154.1 158.8]	[165.2 169.9]
pyruvate kinase	[122.4 126.5]	[150.6 155.8]	[162.2 167.4]
glucose 6-phosphate dehydrogenase	[53.7 55.4]	[28.1 31.2]	[14.5 15.5]
transaldolase	[18.3 18.9]	[9.7 10.7]	[5.1 5.4]
transketolase 1	[18.3 18.9]	[9.7 10.7]	[5.1 5.4]
transketolase 2	[15.9 16.6]	[7.9 9.1]	[3.6 4.0]
pyruvate carboxylase	[21.6 27.0]	[24.5 30.3]	[25.5 37.3]
pyruvate decarboxylase	[60.5 83.8]	[74.4 92.6]	[120.9 126.4]
acetyl CoA synthase	[60.9 84.1]	[10.9 29.3]	[8.5 14.2]
intercompartmental pyruvate transport	[17.8 38.1]	[33.7 51.0]	[7.5 11.2]
intercompartmental oxaloacetate transport	[14.4 19.9]	[18.3 24.9]	[20.8 33.5]
intercompartmental acetyl CoA transport	[41.0 63.6]	[0.0 15.4]	[0.0 2.8]
overall intercompartmental transport	[94.3 100.1]	[65.6 75.7]	[33.2 41.2]
pyruvate dehydrogenase	[9.8 36.5]	[36.2 61.9]	[16.3 31.2]
citrate synthase	[69.3 77.4]	[48.8 60.2]	[17.2 29.4]
oxoglutarate dehydrogenase	[59.2 68.1]	[41.0 53.7]	[10.4 23.9]
malic enzyme	[4.5 10.4]	[10.7 18.3]	[14.0 28.0]
cytosolic alanine synthesis	[0.0 0.0]	[1.0 1.4]	[0.6 1.2]
mitochondrial alanine synthesis	[2.7 3.0]	[0.7 1.1]	[0.5 1.4]
glycine synthesis via serine	[1.5 1.8]	[0.2 1.2]	[1.1 1.5]
glycine synthesis via threonine	[0.5 0.7]	[0.4 1.5]	[0.0 0.4]
NADPH supply by PPP	[107.4 110.8]	[56.2 62.4]	[29.0 31.0]
NADPH demand for anabolism	[92.8 100.8]	[61.7 72.8]	[51.4 64.6]
			
**Flux Reversibility **^b^			
glucose 6-phosphate isomerase	[19.4 25.5]	[21.2 31.9]	[17.6 30.7]
transaldolase	[0.0 0.3]	[0.0 0.3]	[0.0 0.3]
transketolase 1	[2.1 4.0]	[20.7 36.4]	[2.1 2.9]
transketolase 2	[0.0 0.8]	[1.9 4.1]	[0.0 0.2]
intercompartmental oxaloacetate transport	[2.1 3.0]	[0.1 0.4]	[0.9 1.9]
intercompartmental acetyl CoA transport	[17.4 28.6]	[14.8 32.0]	[17.3 30.1]

^a ^The 90 % confidence intervals were obtained by a Monte-Carlo approach including 100 independent parameter estimation runs for each scenario with statistically varied experimental data assuming a normal distribution of the experimental error.

^b ^Flux reversibility is defined as ratio of back flux to net flux.
